# Environmental DNA Metabarcoding Effectively Detects Invasive Species, Pests, and Community Changes in Taiwan's Rice Fields

**DOI:** 10.1002/ece3.73339

**Published:** 2026-04-09

**Authors:** Pritam Banerjee, Gobinda Dey, Kathryn A. Stewart, Matthew A. Barnes, Md. Taharia, Mathew Seymour, Chin‐Wen Wang, Raju Kumar Sharma, Jyoti Prakash Maity, Chien‐Yen Chen

**Affiliations:** ^1^ Department of Environmental Science, Policy, and Management University of California Berkeley California USA; ^2^ Department of Earth and Environmental Sciences National Chung Cheng University Chiayi County Taiwan; ^3^ Institute of Environmental Sciences Leiden University Leiden the Netherlands; ^4^ Department of Natural Resources Management Texas Tech University Lubbock Texas USA; ^5^ School of Biological Sciences The University of Hong Kong SAR Hong Kong SAR China; ^6^ Department of Chemistry, School of Applied Sciences KIIT Deemed to be University Bhubaneswar Odisha India; ^7^ PhD in Doctoral Program in Science, Technology, Environment, and Mathematics, National Chung Cheng University Chiayi Taiwan; ^8^ Center for Nano Bio‐Detection, Center for Innovative Research on Aging Society, AIM‐HI National Chung Cheng University Chiayi Taiwan

## Abstract

Rice fields represent man‐made semi‐aquatic wetlands primed for invasive pests. Monitoring rice field biodiversity using conventional methods, however, is time‐consuming and laborious. Environmental DNA (eDNA) methods can provide a fast and effective means to monitor rice field communities and inform management decisions. Our study provides proof‐of‐concept of rice field eDNA biodiversity assessments, with a focus on native and non‐native pests across cultivation phases. We collected eDNA samples from locations in southern Taiwan rice fields during planting and harvesting time, employing eDNA metabarcoding (COI) to detect diverse taxonomic groups. We assigned 77 ASVs across all sites to animal taxa, 34 of which were identified to species. Overall, 18 species were designated as native or non‐native (83.3% and 16.6%, respectively), including three major rice pests, *Chilo suppressalis* (native), *Coptotermes formosanus* (native), and *Pomacea canaliculata* (non‐native). Cultivation status affected overall diversity, with higher species richness during planting compared to harvesting. No significant differences were observed between native/non‐native taxa and between cultivation phases. Altogether, we detected a complex environment across trophic levels comprised of both native and non‐native agricultural pests using limited sampling effort, demonstrating eDNA analysis as an efficient biomonitoring approach in rice agroecosystems with direct applications for pest, invasive species, and vector surveillance within Taiwan.

## Graphical abstract

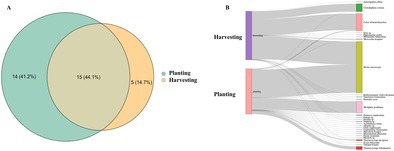

## 1 Introduction

Rice fields represent man‐made, wetland agroecosystems that support a wide range of biodiversity, from microorganisms to macroinvertebrates and amphibians (Bambaradeniya and Amarasinghe 2004). Moreover, their alternating hydrologic dynamics, from flooded during the wet seasons to dry conditions in between, mean rice fields house a unique array of biodiversity (Watanabe et al. 2013). Additional ecological characteristics, such as nutrient‐rich environments, human influences, and the effects of climate change, collectively make these ephemeral wetlands particularly vulnerable to invasive species (Flanagan et al. 2015). Major threats to the ecological stability and biodiversity in the rice fields include anthropogenic pressures (e.g., intensive monoculture, agrochemical use) and the spread of invasive species (Tilman et al. 2002; Horgan 2023).

In Taiwan, rice is a major staple crop, deeply embedded in the economy and culture, with almost half of the arable land consisting of rice fields (Hsing 2014). However, rice fields are one of the most susceptible places for a myriad of invasive pests (Valls et al. 2014). Taiwan's subtropical climate and extensive irrigation networks further exacerbate invasions by non‐native species, posing serious harm to crop productivity and native biodiversity (Barker 2002; Huang et al. 2010; Wu et al. 2010). Several invasive pest species severely impact rice cultivation in Taiwan; for example, the introduction and establishment of *Pomacea* spp. have become serious threats to rice agriculture (Barker 2002; Wu et al. 2010; Banerjee et al. 2022), and are currently responsible for large economic losses throughout Asia (Jiang et al. 2022). Migratory rice planthoppers ( *Nilaparvata lugens* ) (Otuka et al. 2012) and the recently introduced fall armyworm ( *Spodoptera frugiperda* ) (Tsai et al. 2000) also represent major economic concerns. Compounding impacts, native pests, such as rice stem borers (*Chilo suppressalis*, *Sesamia inferens*, and *Scirpophaga incertulas*), planthoppers (e.g., *Sogatella furcifera* ) (Huang et al. 2010), leafhoppers (e.g., *Nephotettix* spp.), and the rice black bug (*Scotinophara coarctata*), additionally contribute to considerable crop damage (Cheng et al. 2010, 2022; Triapitsyn et al. 2021).

Conventional methods of biomonitoring, such as dipnet and funnel traps, are well established and effective for understanding the co‐occurrence of organisms associated with rice cultivation (Song and Kuo 2022), particularly invertebrates. However, conventional methods are also time‐consuming and require taxonomic expertise, limiting the scale and scope needed for establishing large‐scale temporal monitoring needed to enable adaptive resource management (Baird and Hajibabaei 2012). Environmental DNA (eDNA)‐based metabarcoding has recently revolutionized biodiversity monitoring by enabling the non‐destructive detection of targeted or multiple species directly from environmental samples (Taberlet et al. 2012; Valentini et al. 2016; Didaskalou et al. 2022). The adoption of eDNA metabarcoding has proven valuable in agricultural ecosystems, where ecological interactions, including both beneficial and pest organisms, can be monitored for proper management decisions (Kestel et al. 2022; Llanos et al. 2025). However, broad‐scale implementation of eDNA‐based monitoring in agricultural practices remains limited (Kestel et al. 2022) to a few studies assessing general community observations in rice fields (Ushio et al. 2023; Katayama et al. 2024; Vastano et al. 2024). Previous studies have established species‐specific approaches for an early detection method for cryptic invasive gastropods (Banerjee et al. 2022) and fall armyworm (Tsai et al. 2020) in Taiwan. However, more robust and community‐level monitoring is necessary for early management action that goes beyond species‐specific targets.

Recent advancements in high‐throughput sequencing technologies have enhanced the resolution of eDNA metabarcoding approaches, enabling detailed taxonomic and functional assessments of communities present within agricultural environments (Valentini et al. 2016; Seymour et al. 2021; Ushio et al. 2023; Katayama et al. 2024; Vastano et al. 2024). Our study aimed to elucidate a comprehensive understanding of co‐occurring species associated with rice field cultivation across two phases of rice cultivation (i.e., planting and harvesting) in Taiwan. To do so, we implemented an eDNA‐based metabarcoding approach targeting the mitochondrial cytochrome c oxidase I (COI) gene to investigate the community dynamics of native, non‐native, and pest species associated with various rice fields. As environmental conditions (e.g., pH, salinity, temperature, and other abiotic factors) putatively influence the shaping of community composition and function within agroecosystems (Hartmann and Six 2022) and potentially also modulate eDNA detectability and persistence (Barnes and Turner 2016; Stewart 2019), we co‐measured water and soil parameters alongside our eDNA sampling.

We hypothesized that (i) eDNA metabarcoding would be an effective method for detecting the vast array of interactive animal species from rice fields via water samples; and (ii) during the planting season, species diversity would be high, but community composition would demonstrate a higher proportion of colonizing non‐natives and pests compared to harvesting time due to the drier environment.

## 2 Materials and Methods

### 2.1 Sample Collection and Environmental Parameter Analysis

**FIGURE 1:**
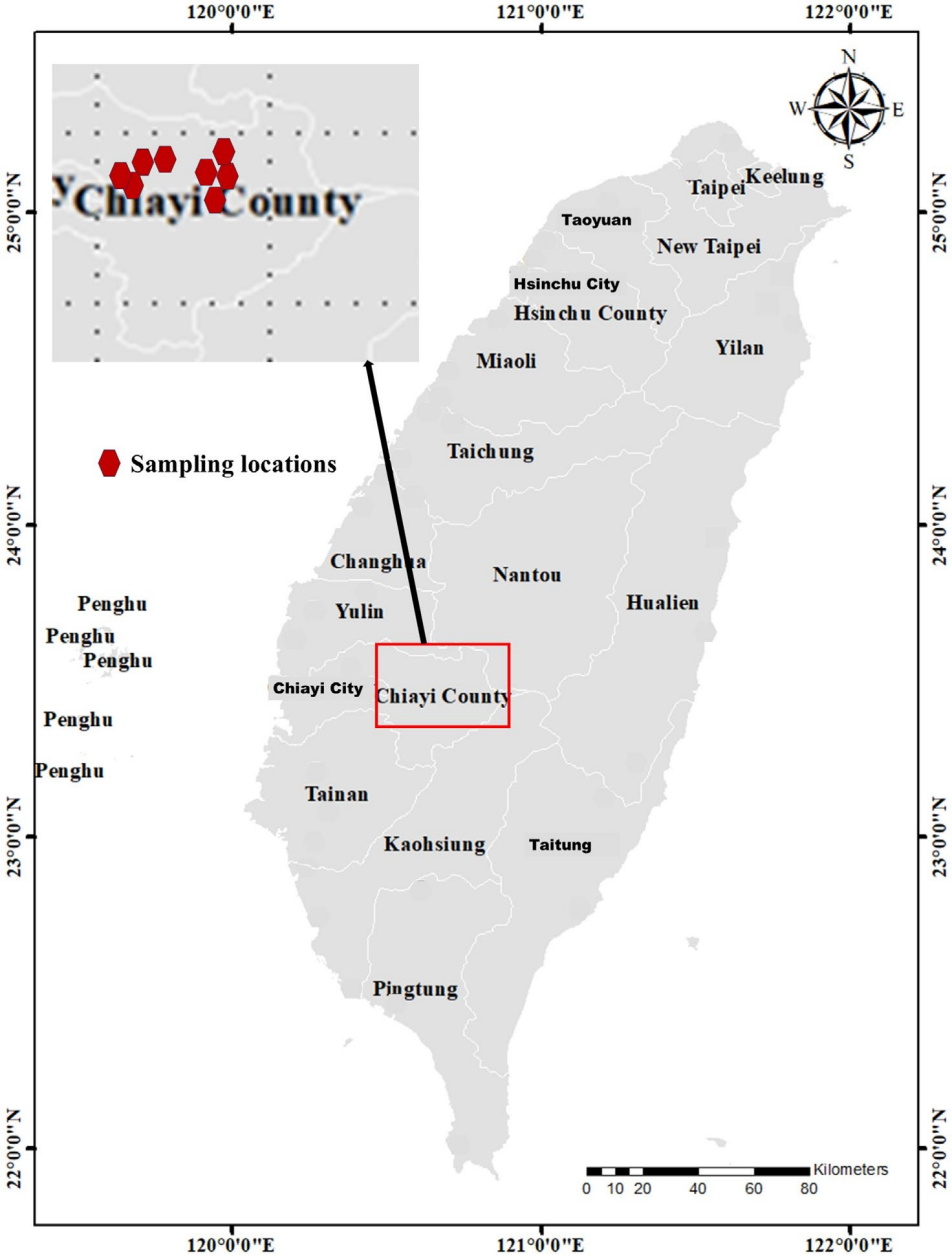
Locations of eight eDNA sampling sites near Chiayi County, Taiwan (February and May 2022).

We collected eDNA samples from eight rice fields in Chiayi County, Taiwan (Figure 1, Table 1). To understand the changes in the community during rice growth, including native and non‐native species, all fields were visited two times: (i) planting phase (denoted as samples “PF1‐PF8”) which consists of the period of time at the beginning of the growing season where crops are seeded and vegetative growth initiates, and (ii) harvesting phase (“HF1‐HF8”), characterized by the ripening phase, with mature rice seedlings and a dry environment. In our study, planting phase sampling occurred on 22 February 2022, and the harvesting phase sampling occurred on 31 May 2022. We collected five 500 mL surface water samples from each field (*N* = 40 samples per season). Water samples were kept on ice and transported directly to the Department of Earth and Environmental Sciences, National Chung Cheng University, for analysis.

**TABLE 1:** Metadata of rice field samples collected at two crop stages.

Sample name	Field type	Location	Date
PF1	Planting	23.567737, 120.454625	10‐Feb‐22
PF2			
PF3		23.540607, 120.380341	
PF4			
PF5		23.567776, 120.454035	
PF6			
PF7		23.543891, 120.384877	
PF8			
HF1	Harvesting	23.567737, 120.454625	31‐May‐22
HF3		23.540607, 120.380341	
HF4			
HF5		23.567776, 120.454035	
HF6			
HF7		23.543891, 120.384877	
HF8			

*Note:* Planting‐stage samples (PF1–PF8) were collected on 10 February 2022 and harvesting‐stage samples (HF1–HF8) were collected on 31 May 2022, from four rice fields located in Chiayi County, Taiwan. Coordinates for each sampling site are provided.

After collection of eDNA, the on site phycochemical properties were measured at each site, including pH, dissolved oxygen (DO) (mg/L), temperature (°C), salinity (PSU), oxidation–reduction potential (ORP) (mV), conductivity (mS/cm), total dissolved solids (TDS) (mg/L), and resistivity (Ω‐cm) using a Hanna multiparameter waterproof meter (HANNA instruments, model HI98194, USA), replicating each measurement ten times per site. Subsequently, the water sample was acidified with 5 M HNO₃ to achieve pH < 2, followed by filtration using 0.22‐μm nylon syringe filters (13 mm; PureTech, Taiwan) into 50‐mL centrifuge tubes for further heavy metals analysis (see the analytical methods below). Additionally, three replicate soil samples (approx. 300 g each) were collected from each site to measure the physicochemical properties, such as pH and texture (percentage of sand, silt, and clay) as well as heavy metals concentration, following protocols outlined in Dey et al. (2022). Soil pH was measured using a 1:2.5 soil‐to‐deionized water suspension with a pH meter. Soil particle size distribution was analyzed by the hydrometer method and classified into textural classes following USDA‐NRCS guidelines (Bouyoucos 1962).

Concentrations of elements (Ag, Bi, Li, Cd, Mn, Cu, Ni, Co, Cr, Sr., Zn, Pb, and Fe) in filtered acid‐digested water and soil samples were analyzed using Inductively Coupled Plasma Optical Emission Spectrometry (ICP‐OES; Agilent Technologies 5100 ICP‐OES, CA, USA). To ensure accuracy and precision, a negative control ultrapure water (PP1‐185UV Type I, UNISS, Taiwan) and a positive reference standard were included (ICP multi‐element standard solution IV). The recovery rates for the analyzed metals ranged between 94% and 115%, with precision maintained under a 10% relative standard deviation (RSD) for all elements. The soil samples were dried at 70°C for 48 h, followed by grinding in a mortar and pestle, and then were prepared for heavy metal concentrations analysis using ICP‐OES (Agilent Technologies 5100 ICP‐OES, USA), based on our previously standardized method described in Dey et al. (2024). Briefly, 0.4 g dried soil was digested with 8 mL aqua regia (a 3:1 mixture of HCl and HNO3) and 2 mL 40% hydrofluoric acid (HF). After cooling, the solution was diluted with 30 mL of deionized water and then filtered with 0.22 μm nylon syringe filters (13 mm; PureTech, Taiwan) before the ICP‐OES analysis.

### 2.2 eDNA Filtration, Extraction, and Amplification

In the laboratory, all water samples were passed through a GN‐6 Metricel membrane mixed cellulose ester filter paper (GN‐6 Metricel, Pall Corporation, USA) with a diameter of 47 mm and a pore size of 0.45 μm. As rice field water has high TDS, any clogging issues were mitigated by using multiple filter papers as sub‐samples (e.g., Hunter et al. 2019). Once our filtration was complete, half of each filter paper was processed for immediate eDNA extraction, and the latter half was preserved at −20°C in Longmire's buffer for future use (Longmire et al. 1997). For samples that required multiple sub‐filters to mitigate clogging, all filter papers corresponding to the same sample were placed in a single tube for DNA extraction. During the filtration process, three filtration blanks (distilled water) were included to determine contamination and false positive detection during laboratory analysis. Sterilization processes were maintained throughout the workflow, like (i) frequent decontamination with 70% ethanol and 10% bleach, (ii) unidirectional workflow with each step (i.e., filtration, DNA extraction, PCR, and post‐PCR) performed in a separate space, and (iii) PCRs were performed in a well‐maintained PCR workstation.

We extracted eDNA from filters using DNeasy Blood & Tissue kits (Qiagen, Germany) according to the manufacturer's protocol, but with some modifications as follows: (i) the filter papers were incubated at 60°C overnight in a mixture of 180 μL of ATL lysis buffer and 20 μL Proteinase K; and (ii) the elution step was performed twice, where the spin columns were incubated with the same 50 μL elution buffer at 37°C for 10 min (Spens et al. 2017). The quality and quantity of extracted DNA samples were assessed using a Nanodrop spectrophotometer (Implen, Germany) and an Invitrogen Qubit 3.0 fluorometer (Thermo Fisher Scientific, USA). For budgetary reasons, a pooling approach was used for sequencing (Melcher et al. 2024). After extractions, all subsamples (5 samples from each field) were pooled together based on their quantity. Thus, making 8 pooled samples for each planting (*n* = 8) and harvesting (*n* = 8). Finally, these 16 pooled samples were used for further downstream analysis.

A portion of mitochondrial cytochrome c oxidase subunit I (COI) gene was amplified with the following primers: mlCOIintF‐5′‐GGWACWGGWTGAACWGTWTAYCCYCC‐3′ and jgHCO2198‐5′‐TAIACYTCIGGRTGICCRAARAAYCA‐3′, specially designed for metazoans (Leray et al. 2013). The reaction mixture of 25 μL for conventional PCR was prepared by mixing 5 μL 5× Fast‐Run Taq Master Mix with Dye (Protech Technology Enterprise, Taiwan), 0.5 μL each 10 pmol forward and reverse primers (Genomics, Taiwan), 3 μL eDNA sample, and adding high‐quality sterile distilled water (Thermo Fisher Scientific, USA) to make up the final volume. Three PCR replicates for each sample (including blank samples) were carried out. The PCR reactions were performed with the following conditions: an initial denaturation at 94°C for 5 min, followed by a continuous amplification process of 36 cycles, with denaturation at 94°C for 30 s, annealing at 55°C for 30 s, and extension at 72°C for 1 min. The final extension was at 72°C for 5 min after. The PCR products were visualized in a 1.5% agarose gel electrophoresis, and all subsamples/replicates were mixed in equal density ratios (based on band intensity) using the Gel Doc XR system with the QUANTITY ONE software (Bio‐Rad, USA). No visible amplification was observed in any blank sample (filtration or extraction blanks); these were therefore excluded from further analysis. The PCR products were then purified using 1.5× SPRI beads (AMPure XP; Beckman Coulter, USA). Purified amplicons were quantified using an Invitrogen Qubit 3.0 fluorometer and submitted for library preparation and sequencing at Genomics Bioscience and Technology Co. Ltd., Taiwan.

### 2.3 Library Preparation and Next‐Generation Sequencing

Libraries were prepared using the TruSeq Nano DNA Library Prep Kit (Illumina, USA), and quality was assessed using an Agilent Bioanalyzer 2100 system (Agilent Technologies, USA). The target inserts (~313 bp of the mitochondrial COI gene fragment) were derived from the PCR amplification described earlier. The final library size, including Illumina adapters, was approximately 450–500 bp, as measured by Bioanalyzer. Libraries were individually indexed during preparation and pooled (multiplexed) in equimolar concentrations before sequencing. Library quantification was performed using qPCR to ensure accurate equimolar pooling. All qualified libraries were sequenced using 300 bp paired‐end reads on an Illumina MiSeq platform (Illumina, USA), operated by Genomics Bioscience and Technology Co. Ltd., Taiwan. Sequencing was carried out on a multiplexed standard v3 MiSeq flow cell.

### 2.4 Data Processing

Raw sequencing data were demultiplexed using an in‐house script developed by Genomics Bioscience and Technology Co. Ltd., Taiwan, to assign reads to their corresponding samples based on barcode sequences. Trimmomatic (v0.39) was used to remove the adapter sequences, and low‐quality bases (QV < 3) using with the following parameters: ILLUMINACLIP:adapter:2:30:10:1:true LEADING:3 TRAILING:3 SLIDINGWINDOW:4:15. This configuration removes Illumina adapter sequences (allowing up to two seed mismatches), trims low‐quality bases from both ends of the reads, and applies sliding window trimming when the average Phred quality score within the window falls below 15 (Bolger et al. 2014). Sequences corresponding to both ends of the mlCOIintF and jgHCO2198 primers were trimmed using Cutadapt (v3.4) (Martin 2011). Primer trimming was performed with a minimum read length cutoff of 150 bp to remove short, low‐information reads, and reads lacking primer sequences were discarded to ensure that only primer‐containing sequences were retained. The trimming allowed a minimum overlap of 3 bp and an error rate of 0.1 for primer matching. Denoising, chimera removal, and assignment to Amplicon Sequence Variants (ASVs) were performed using DADA2 (Callahan et al. 2016), following the approach of (Sigsgaard et al. 2021). After error removal, all remaining sequences were then compared against the GenBank nucleotide database (Sayers et al. 2022) (as of September 2022) using BLASTN, and taxa were selected based on the Lowest Common Ancestor (LCA) method (https://github.com/timkahlke/BASTA). The criteria for sequence alignment included a maximum of 500 aligned sequences per query, with a ≥ 90% query cover and 70%–100% sequence similarity. For taxonomic assignments, we applied similarity thresholds as follows: 80%–84% for class‐level, 85%–89% for order‐level, 90%–94% for family‐level, 95%–97% for genus‐level, and > 98% for species‐level (Sigsgaard et al. 2021). In cases where multiple taxonomic matches occurred at a given level, the ASV was assigned to the next higher taxonomic rank. The species detected via eDNA metabarcoding were classified as native or non‐native based on previous literature and the Catalog of Life in Taiwan (https://taicol.tw/). Furthermore, the ecological roles were documented from previously published literature accessed through Google Scholar, PubMed, and Web of Science.

### 2.5 Statistical Analysis

All statistical analyses were performed using R version 4.3.3 (R Core Team 2025). Alpha diversity was estimated as observed ASV richness (number of unique ASVs per sample) and visualized across field types using *ggplot2*. Differences in richness between cultivational phases were assessed using Wilcoxon rank‐sum tests. Beta‐diversity was assessed using non‐metric multidimensional scaling (NMDS) based on Bray–Curtis dissimilarities with the metaMDS() function in the vegan package (Oksanen et al. 2001). Group differences in community composition between cultivational stages were tested using PERMANOVA (*adonis2*). To visualize the associations between species and cultivation phases (harvesting and planting), a sankey diagram was constructed using the *networkD3* package (Allaire et al. 2014). All visualization of taxonomic distributions and sample overlaps were performed using the *ggplot2* and *eulerr* packages (Wickham 2016; Yan 2021). For the environmental parameter, mean values ± standard error (SE) were calculated, and differences between harvesting and planting periods were evaluated using Welch's two‐sample *t*‐test (*α* = 0.05).

## 3 Results

### 3.1 Community‐Level Dynamics of Rice Fields

#### 3.1.1 Sequencing Reads and Taxonomic Composition

After filtering, we discarded one sample (HF2) due to low sequencing reads (< 3000). Overall, 1,014,225 sequencing reads were retained, resulting in 67,615 ± 10,984 reads (mean ± standard error; *n* = 15) per sample. Overall, 196 amplicon sequence variants (ASVs) were initially identified across taxa, including Metazoa, Protozoa, Algae, Fungi, and Bacteria. Of the total sequence reads, 54.92% originated from Animalia (80 ASVs), 12.16% from Protozoa (25 ASVs), 4.24% from Bacteria (13 ASVs), 3.29% from Algae (38 ASVs), and 1.40% from Fungi (38 ASVs) (Table 2). Additionally, 23.95% of reads could not be taxonomically assigned with high confidence, as they fell below the 80% similarity threshold (2 ASVs) (Table 2). After removing unassigned taxa (2 human ASVs were also removed) and targeting animal taxa only (one ASV that could identify up to Animalia was removed), a total of 77 ASVs were retained for downstream analysis. The majority of reads (93.12%) were from Arthropoda (43 ASVs), followed by Annelida (11 ASVs), Rotifera (8 ASVs), Mollusca (5 ASVs), Chordata (4 ASVs), Gastrotricha (2 ASVs), Cnidaria (2 ASVs), and Nematoda (2 ASVs) (Table 3). In total, 34 species were identified from various groups, and among them, 18 species could be designated as either native or non‐native based on published literature, involving 83.3% native and16.6% non‐native (Table 4).

**TABLE 2:** Taxonomic distribution of amplicon sequence variants (ASVs) across major groups.

Phylum	Total reads	ASVs	Percentage of reads
Animalia	557,061	80	54.92
Below 80	242,931	2	23.95
Protozoa	123,431	25	12.16
Bacteria	43,090	13	4.24
Algae	33,448	38	3.29
Fungi	14,264	38	1.40

*Note:* The table summarizes the total number of sequencing reads, ASV richness, and percentage of reads assigned to each group. The majority of reads were assigned to Animalia, followed by unclassified reads with < 80% similarity (“Below 80”), Protozoa, Bacteria, Algae, and Fungi.

**TABLE 3:** Distribution of Animalia amplicon sequence variants (ASVs) across phyla.

Phylum	Total reads	ASVs	Percentage of reads
Arthropoda	518,717	43	93.12
Annelida	31,958	11	5.73
Rotifera	3257	8	0.58
Mollusca	1614	5	0.28
Gastrotricha	779	2	0.13
Cnidaria	355	2	0.06
Nematoda	108	2	0.01
Chordata	53	4	~0.01

*Note:* The table shows the total number of sequencing reads, ASV richness, and relative percentage of reads assigned to each phylum. Arthropoda was the dominant phylum, followed by Annelida and Rotifera, while other phyla were represented at much lower abundances.

**TABLE 4:** Taxonomic composition, ecological roles, and native/non‐native status of organisms detected in rice field ecosystems.

Phylum	Species	Ecological role	Status
Annelida	*Metaphire posthuma*	Detritivores	Non‐native (Tsai et al. 2000)
	*Allonais* sp.		N/A
	*Bothrioneurum vejdovskyanum*		N/A
	*Dero* sp.		N/A
	*Pristina* sp.		N/A
Arthropoda	*Ceriodaphnia cornuta*	Primary consumer	N/A
	*Daphnia* sp.	Primary consumer	N/A
	*Moina macrocopa*	Primary consumer	N/A
	*Homidia socia*	Detritivore	Native (Cheng et al. 2022)
	*Thermocyclops decipiens*	Secondary consumer	Native (Reid 1989)
	*Thermocyclops taihokuensis*	Secondary consumer	Native (Reid 1989)
	*Coptotermes formosanus*	Detritivores	Native (Blumenfeld et al. 2021)
	*Tanypus kraatzi*	Detritivores, and Omnivorous	NA
	*Tanytarsus formosanus*	Detritivores, and Omnivorous	NA
	*Culex tritaeniorhynchus*	detritivore/filter‐feeder (larva) and hematophagous (adult)	Native (Chung et al. 2024)
	*Psilopa* sp.	Detritivores	N/A
	*Sciara humeralis*	Detritivores (Larva), feed on nectar or fungi (adult)	Native
	*Diplonychus esakii*	Predator	Native (https://taicol.tw/en‐us/)
	*Microvelia douglasi*	Predator	Native (Ye et al. 2014)
	*Monomorium pharaonis*	Omnivorous	Native (Wetterer 2010)
	*Pheidole* sp.	Omnivorous	N/A
	*Chilo suppressalis*	Primary consumer (Rice crop pest)	Native (Chu 1991)
Chordata	*Microhyla fissipes*	Predator	Native (Jang‐Liaw and Chou 2015)
	*Fejervarya* sp.	Predator	N/A
	*Passer montanus*	Omnivorous	Native (Ding et al. 2020)
	*Acridotheres tristis*	Omnivorous	Non‐native (Ding et al. 2020)
Gastrotricha	*Chaetonotus* sp.	Detritivores, microphagus	N/A
Mollusca	*Pomacea canaliculata*	Detritivorous herbivores. Primary consumer, Rice crop pest	Non‐native (Banerjee et al. 2024)
	*Austropeplea ollula*	Detritivorous herbivores. Primary consumer	Native (Chang 1991)
	*Melanoides tuberculata*	Detritivorous herbivores. Primary consumer	Native (Chang 1991)
Rotifera	*Brachionus* sp.	Detritivores	N/A
	*Keratella* sp.		
	*Lecane* sp.		
	*Polyarthra* sp.		
	*Rotaria* sp.		

*Note:* Species are categorized by phylum, with ecological roles spanning primary consumers, detritivores, predators, and omnivores. Status is assigned based on literature references, indicating whether species are native, non‐native, or not available (N/A).

#### 3.1.2 Environmental Parameters

Soil pH was slightly alkaline (7.74 ± 0.05 at harvesting, 7.54 ± 0.07 at planting) across sites (Table 5), with textures ranging from sandy loam to loamy sand. Particle size composition remained consistent across both cultivation times, dominated by sand (approximately 72%). Heavy metal concentrations in soil were found to exhibit temporal variation, with Cd, Co, Cr, Cu, Ni, Zn, and Ag generally higher during harvesting, though differences were not statistically significant (Table 5). In contrast, both pH and Lead (Pb) were significantly elevated in soil at harvest (*p* < 0.05; Table 5).

**TABLE 5:** Physiochemical properties and heavy metals concentration in agricultural soil during harvesting and planting.

Parameter	Harvesting (Mean ± SE)	Planting (Mean ± SE)	*p* (Welch *t*‐test)
pH	7.74 ± 0.05 ^a^	7.54 ± 0.07 ^b^	**0.0394**
Silt (%)	14.53 ± 1.69 ^a^	15.15 ± 1.59 ^a^	0.8075
Clay (%)	13.10 ± 1.65 ^a^	12.80 ± 1.47 ^a^	0.9016
Sand (%)	72.37 ± 1.63 ^a^	72.05 ± 1.46 ^a^	0.8937
Cd (mg/kg)	0.44 ± 0.07 ^a^	0.30 ± 0.06 ^a^	0.1694
Co (mg/kg)	2.41 ± 0.57 ^a^	2.02 ± 0.35 ^a^	0.6029
Cr (mg/kg)	70.94 ± 6.48 ^a^	64.66 ± 7.12 ^a^	0.5536
Cu (mg/kg)	19.40 ± 1.77 ^a^	16.46 ± 2.10 ^a^	0.3379
Fe (mg/kg)	29781.25 ± 2729.60 ^a^	24943.06 ± 2848.57 ^a^	0.2743
Li (mg/kg)	37.34 ± 2.49 ^a^	37.73 ± 1.77 ^a^	0.9074
Mn (mg/kg)	256.67 ± 32.80 ^a^	243.13 ± 36.91 ^a^	0.8026
Ni (mg/kg)	35.11 ± 2.85 ^a^	32.98 ± 3.93 ^a^	0.6899
Pb (mg/kg)	35.81 ± 1.39 ^a^	29.04 ± 2.49 ^b^	**0.0492**
Sr (mg/kg)	269.93 ± 34.18 ^a^	306.87 ± 27.48a	0.4489
Zn (mg/kg)	92.91 ± 9.04 ^a^	86.12 ± 7.95^a^	0.6092
Ag (mg/kg)	1.26 ± 0.15 ^a^	1.14 ± 0.21^a^	0.6702

*Note:* *p*‐value are from Welch's two‐sample *t*‐test (*α* = 0.05), a and b subscripts represent significant differences, and bolded *p‐values* in *p* (Welch *t*‐test) indicate statistical significance.

Water parameters also reflected alkaline pH (7.52 ± 0.05 at harvesting, 7.54 ± 0.05 at planting). ORP was significantly higher during the planting phase (188.99 ± 3.77) compared to the harvesting phase (152.37 ± 11.67; *p* = 0.0270). Dissolved oxygen (DO) was also higher during planting (5.27 ± 0.26 mg/L) than harvesting (4.45 ± 0.02 mg/L; *p* = 0.0197) (Table 6). Similarly, conductivity increased during planting (0.55 ± 0.05 mS/cm) relative to harvesting (0.40 ± 0.04 mS/cm; *p* = 0.0363), and total dissolved solids (TDS) were greater in planting (259.80 ± 22.12 mg/L) than harvesting (187.08 ± 20.39 mg/L; *p* = 0.0424). In contrast, temperature was significantly higher during the harvesting phase (29.32°C ± 0.88°C) compared to the planting phase (26.08°C ± 0.76°C; *p* = 0.0231). Among trace metals, only lithium (Li) showed a statistically significant difference, with slightly higher values during harvesting (0.27 ± 0.00 mg/L) than planting (0.26 ± 0.00 mg/L; *p* = 0.0082). Other parameters, including pH, mV pH, resistivity, PSU, atmospheric pressure, Fe, Mn, and Sr., did not differ significantly between phases (*p* > 0.05) (Table 5).

**TABLE 6:** The physicochemical properties and heavy metals concentration of water at agricultural fields during harvesting and planting.

Parameter	Harvesting (Mean ± SE)	Planting (Mean ± SE)	*p* (Welch *t*‐test)
pH	7.52 ± 0.06 ^a^	7.54 ± 0.05 ^a^	0.7818
mV pH	−21.48 ± 2.63 ^a^	−26.70 ± 2.52 ^a^	0.2059
ORP	152.37 ± 11.67 ^b^	188.99 ± 3.77 ^a^	**0.0270**
DO (mg/L)	4.45 ± 0.02 ^b^	5.27 ± 0.26 ^a^	**0.0197**
Conductivity (mS/cm)	0.40 ± 0.04 ^b^	0.55 ± 0.05 ^a^	**0.0363**
Resistivity (ohm·cm)	3070.52 ± 382.50 ^a^	2111.05 ± 220.36 ^a^	0.0723
TDS (mg/L)	187.08 ± 20.39 ^b^	259.80 ± 22.12 ^a^	**0.0424**
PSU	0.26 ± 0.02 ^a^	0.25 ± 0.02 ^a^	0.7992
Temperature (°C)	29.32 ± 0.88 ^a^	26.08 ± 0.76 ^b^	**0.0231**
Atm. Pressure (PSI)	14.57 ± 0.10 ^a^	14.76 ± 0.02 ^a^	0.1412
Fe (mg/L)	0.15 ± 0.07 ^a^	0.03 ± 0.01 ^ab^	0.1676
Li (mg/L)	0.27 ± 0.00 ^a^	0.26 ± 0.00 ^b^	**0.0082**
Mn (mg/L)	0.31 ± 0.10 ^a^	0.24 ± 0.11 ^a^	0.6609
Sr (mg/L)	3.90 ± 0.87 ^a^	6.61 ± 1.09 ^a^	0.0938

*Note:* *p*‐value are from Welch's two‐sample *t*‐test (α = 0.05). a and b subscripts represent significant differences, and bolded *p‐values* in *p* (Welch *t*‐test) indicate statistical significance.

#### 3.1.3 Planting Versus Harvesting Phase

**FIGURE 2:**
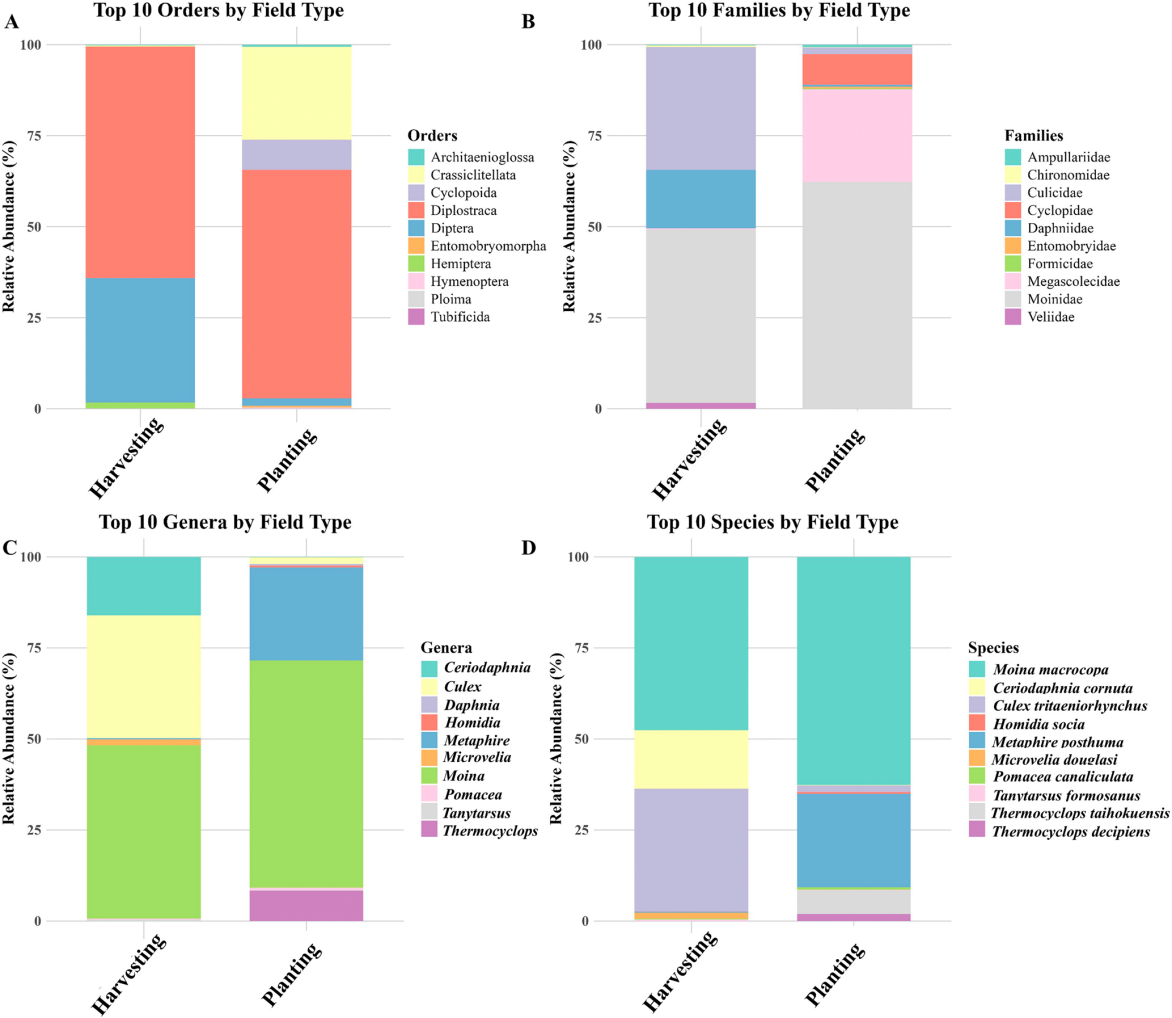
Relative abundance of the top metazoan 10 taxa by field type. Stacked bar plots showing the relative abundance (%) of the top 10 taxa detected in *harvesting* and *planting* field types, displayed at four taxonomic levels: (A) orders, (B) families, (C) genera, and (D) species.

The relative abundance of the top 10 taxa at different taxonomic levels (order, family, genus, and species) varied markedly between harvesting and planting phases (Figure 2). At the order level, Diplostraca was the dominant order in both field types, accompanied primarily by Diptera in harvesting fields and by Crassiclitellata and Cyclopoida in planting fields. At the family level, Moinidae dominated both harvesting and planting fields, co‐occurring with Culicidae and Daphniidae in harvesting fields and with Megascolecidae and Cyclopidae in planting fields. At the genus level, *Moina* sp. dominated both harvesting and planting fields, co‐occurring with *Culex* sp. and *Ceriodaphnia* sp. in harvesting fields and with *Metaphire* sp. and *Thermocyclops* sp. in planting fields. At the species level, *Moina macrocopa* was dominant in both fields, accompanied by *Culex tritaeniorhynchus* and *Ceriodaphnia cornuta* in harvesting fields and by *Metaphire posthuma* in planting fields.

**FIGURE 3:**
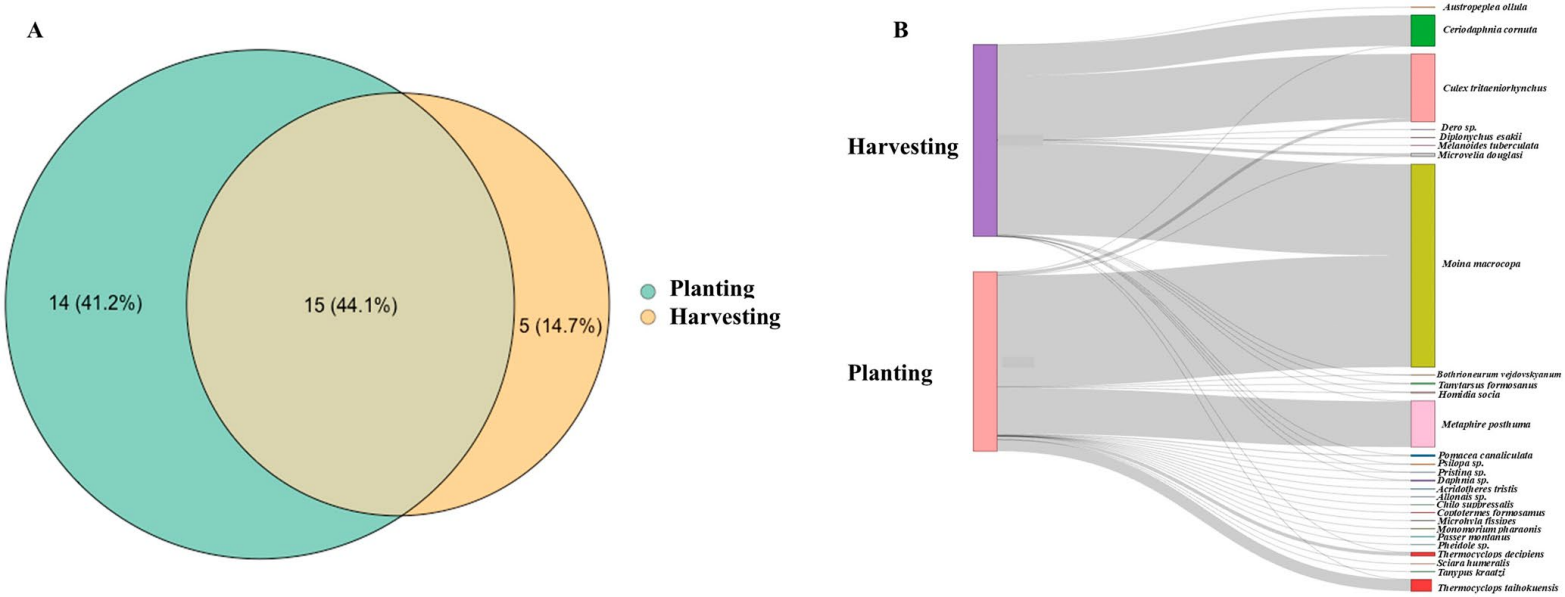
(A) Venn diagram of species detected in planting and harvesting fields. Circle and overlap size denote proportional species diversity representation across field types. (B) Sankey diagram showing the association between rice field types (planting and harvesting) and detected species.

**FIGURE 4:**
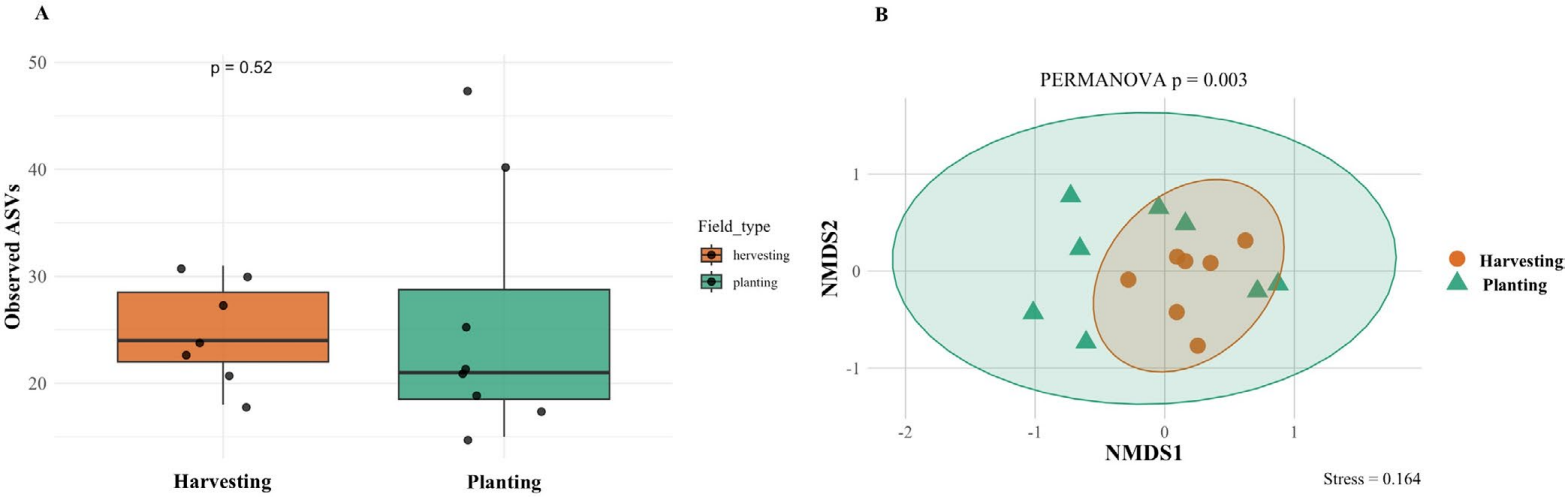
(A) Comparison of alpha diversity (observed ASVs) between field types (harvesting, orange; planting, green) across sites (*n* = 15). (B) Non‐metric multidimensional scaling (NMDS) plot based on Bray–Curtis dissimilarity showing beta diversity of communities between planting (green) and harvesting (orange) rice fields detected from eDNA metabarcoding.

**FIGURE 5:**
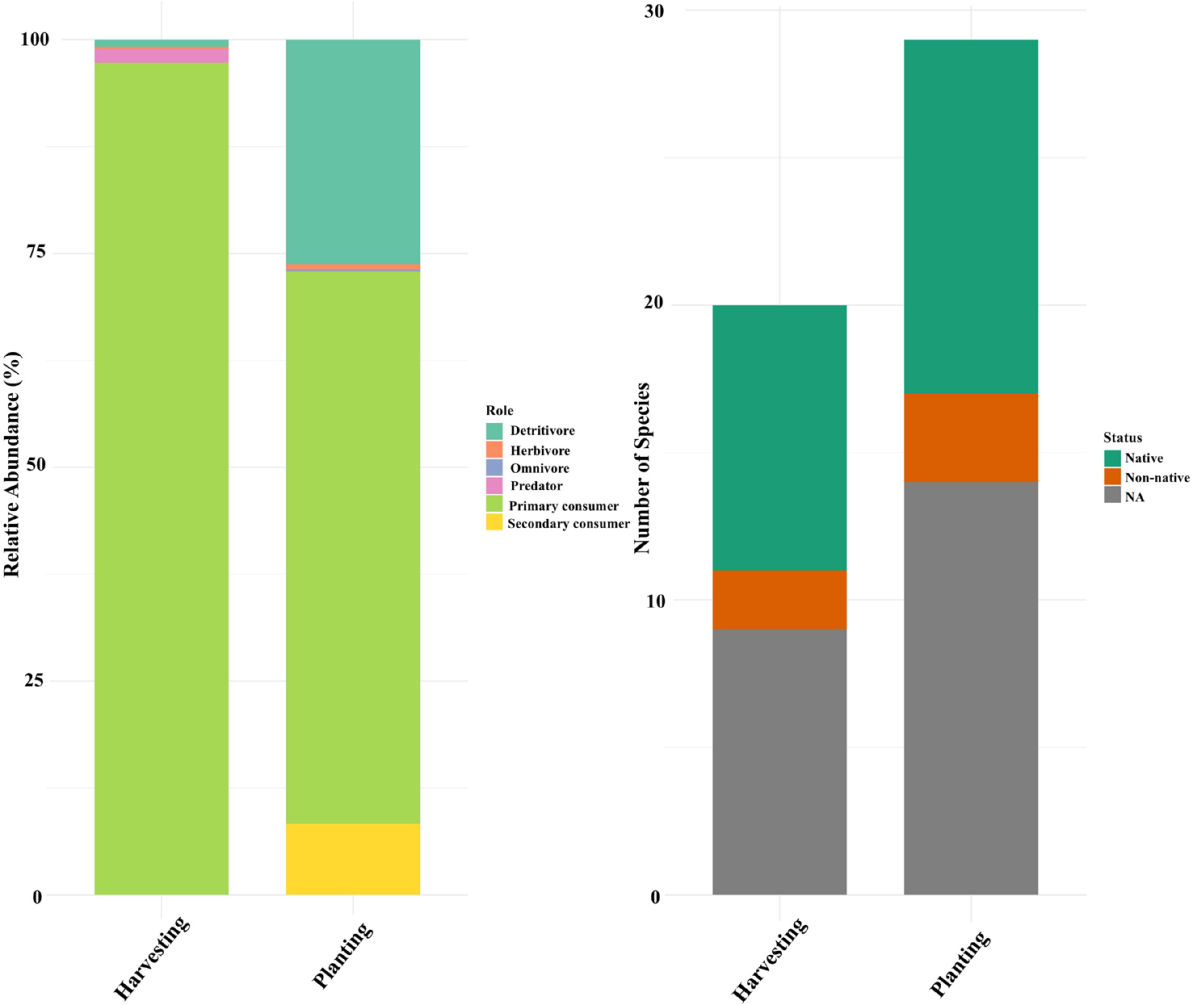
Functional roles and species status across rice field types. (Left) Relative abundance of species categorized by functional roles during harvesting and planting. (Right) Species richness grouped by published literature and the Catalog of Life in Taiwan (https://taicol.tw) (Table 4; native, non‐native, and undermined/NA).

Of note, species overlap between planting and harvesting stages was substantial (44.1%; Figure 3A) but also demonstrated some exclusive taxa between both cultivation phases, with more unique species during the planting (35.7%) than the harvesting phase (14.3%) (Figure 3A,B). This was corroborated by a Sankey diagram showing the association between the cultivation phases and the relative abundance (based on sequencing reads) of taxa (Figure 3B). Moreover, species richness was higher during the planting phase, with 12 native, 3 non‐native, and 14 undetermined (status) taxa, compared to 9 native, 2 non‐native, and 9 undetermined (status) taxa in the harvesting phase (Figure 4, Table 4). Still, alpha‐diversity did not differ significantly between harvesting and planting phases (*p*‐value = 0.52) (Figure 5A). The median species richness was slightly higher during harvesting compared to planting phases, although the variation was larger during planting, with a few high diversity outliers (Figure 5A).

Despite no significant differences in alpha‐diversity across cultivation times, the non‐metric multidimensional scaling (NMDS) showed a pattern of differing community composition between cultivation times (Figure 5B) (stress = 0.164). The PERMANOVA test confirmed that the difference between the two growth phases was statistically significant (*p* = 0.003). Additionally, no significant differences were observed between native and non‐native taxa across the two cultivation phases (Wilcoxon test, *p* > 0.99).

## 4 Discussion

In this study, we aimed to establish eDNA metabarcoding as an effective method for detecting the vast array of interactive animal species from rice fields. Overall, the eDNA‐based method successfully detected a wide range of taxonomic groups during both planting and harvesting phases, even with minimal sampling time and effort (Figure 3B, Table 4), consistent with the first hypothesis (H1). We successfully detected rice pests, including *Chilo suppressalis* (native), *Coptotermes formosanus* (native), and *Pomacea canaliculata* (non‐native), among others (Table 4). Our results indicate that changes in rice cultivation phases correspond to significant changes in community composition (Figure 3A,B), though the species richness between the phases remains relatively static (Figure 5A). We didn't observe any significant difference between native and non‐native taxa across the two cultivation phases (Figure 4), counter to our second hypothesis (H2). However, this may also be a consequence of the relatively small sample size and limited study area. Future studies incorporating a larger number of samples and broader spatial coverage would improve the robustness of the analysis and may reveal patterns that were not detectable in the present study.

### 4.1 Functional Role of the Detected Organism

Our analysis of the rice field ecosystem revealed a diverse community spanning multiple trophic levels, including primary consumers, detritivores, predators, omnivores, and native and non‐native agricultural pests (Table 4). Primary producers included rice crops and various phytoplankton. Detritivores such as *Metaphire posthuma* (Tsai et al. 2020), aquatic oligochaetes (*Allonais*, *Dero*, *Pristina*), soil invertebrates (e.g., *Homidia socia*, *Coptotermes formosanus* ) (Cheng et al. 2022; Blumenfeld et al. 2021), and gastropods (*Melanoides tuberculata*) have an important role in nutrient cycling (Chang 1991). Phytoplankton grazers included cladocerans (e.g., *Moina macrocopa* , *Ceriodaphnia cornuta* ), rotifers (*Brachionus*, *Keratella*), and gastropods ( *Pomacea canaliculata* , *Austropeplea ollula*) (Table 4). Omnivores such as ants ( *Monomorium pharaonis* , *Pheidole* sp.) (Wetterer 2010), and birds ( *Passer montanus* , *Acridotheres tristis* ) were also detected (Ding et al. 2020). In this context, birds may play a potential role as insect predators (Sottomayor et al. 2024). Secondary consumers included *Thermocyclops decipiens* and *T. taihokuensis* (Reid 1989), while predators such as frogs ( *Microhyla fissipes* , *Fejervarya* sp.) (Jang‐Liaw and Chou 2015) and aquatic insects ( *Diplonychus esakii* , *Microvelia douglasi* ) further structured the food web (Ye et al. 2014).

Two taxa of particular concern, *Chilo suppressalis* and *Pomacea canaliculata* , are major rice pests responsible for economic damage (Chu 1991); however, the detection of *Coptotermes formosanus* is also a concern, as it may damage the rice plants (Li et al. 2009). We also recorded high abundances of *Culex tritaeniorhynchus* (Figure 2), a vector of Japanese encephalitis virus, which may raise concerns about the management of stagnant water in rice fields (Table 4).

### 4.2 Planting Versus Harvesting Phase

The NMDS results demonstrate that arthropod community structure differs significantly between harvesting and planting phases. The tighter clustering of harvesting compared to the planting phase suggests more homogeneous community assemblages under harvesting conditions, possibly due to low availability of water or selective pressures associated with crop removal (Flannagan and Marshall 2012; Saksongmuang et al. 2024). In contrast, the broader spread of identified ASVs during planting phases indicates greater community heterogeneity, which may reflect colonization dynamics, variability in early vegetation structure, or differences in resource availability (Mukherjee and Khan 2017; Saksongmuang et al. 2024). This pattern is also supported by the measured physicochemical parameters, with the planting phase showing higher DO, ORP, conductivity, and TDS compared to the harvesting phase, possibly due to longer water residence time and more biological activity, whereas the harvesting stage was characterized by relatively drier conditions and reduced water availability (Table 6). Among the analyzed elements in the water samples, only Fe, Li, Mn, and Sr were detected above the method detection limit. However, in this study, the relationship between environmental parameters and indicator taxa could be better resolved with more robust seasonal sampling.

The significant PERMANOVA results support our proposition that these biodiversity compositional differences are not random but instead driven by the phase of rice cultivation. This may highlight that agricultural practices, even across short time scales, can influence community composition, possibly through habitat modification, disturbance regimes, and resource distribution (Mukherjee and Khan 2017). The harvesting phase encompasses warmer conditions compared to the planting phase, driven by warmer season and slight increases in soil pH and Pb, probably due to cumulative agrochemical deposition.

### 4.3 Native and Non‐Native Status

Two frog species were identified. Among these, *Microhyla fissipes* is recognized as native and widely distributed (Jang‐Liaw and Chou 2015). The other taxon, *Fejervarya* sp., was identified only to the genus level, which includes both a native species ( *Fejervarya limnocharis* ) and a non‐native species ( *Fejervarya cancrivora* ) present in Taiwan (Jang‐Liaw and Chou 2015; Lee et al. 2019). One native ( *Passer montanus* ) and an introduced bird ( *Acridotheres tristis* ) with widespread distribution in Taiwan were also detected from the rice field water (Ding et al. 2020). Among arthropods, *Coptotermes formosanus* is reported as a native pest species (Li et al. 2009; Blumenfeld et al. 2021); however, this species is considered among the top 100 invasive species in the world, and its introduced population has been established in Japan, Hawaii, and the southeastern United States (Simberloff and Rejmanek 2019; Blumenfeld et al. 2021). The native arthropod taxa detected included *Homidia socia*, *Culex tritaeniorhynchus*, *Sciara humeralis*, *Diplonychus esakii* , *Microvelia douglasi* , *Monomorium pharaonis* , and *Chilo suppressalis*. The non‐native earthworm *Metaphire posthuma* was detected (Tsai et al. 2000); this species is now naturalized and does not appear to negatively impact the local environment. Additionally, one invasive snail ( *Pomacea canaliculata* ), responsible for various crop damage, and two native snails (*Austropeplea ollula* and *Melanoides tuberculata*), were detected together in the rice fields (Chang 1991).

Although eDNA analysis of rice field water provided a comprehensive assessment of species presence, the array of biodiversity detection was still less than previous conventional surveys conducted in Taiwan (Song and Kuo 2022; Chen et al. 2023). In our results, we detected 77 animal ASVs, of which 34 were assigned to species level, which is lower than reported in previous conventional studies (Song and Kuo 2022; Chen et al. 2023). The disparity with other studies may be due to lower sampling effort (16 pooled samples) in this study, where repetitive monitoring and an increase in sampling events can increase the detection (Yates et al. 2023). We did not explore the effects of eDNA persistence dynamics under hot climatic conditions and potential PCR inhibition (McCartin et al. 2022), but their influence cannot be neglected. Furthermore, universal concerns related to primer bias and limitations of reference databases require further investigation (Korbel et al. 2024). Taiwan has a good reference library (Hu et al. 2024), a direct comparison between the conventional detection and eDNA analysis can collect more evidence of disparity. Thus, future studies should implement a comparative analysis of conventional and eDNA methods to understand the potential community shift during cultivation and the robustness of eDNA biomonitoring compared to conventional approaches.

Fewer detections with eDNA sampling compared to conventional approaches may also suggest that the water sampling volume used here might not be sufficient to capture broad diversity (Cantera et al. 2019). Frequent clogging of filter papers from eDNA in turbid habitats, such as wetlands, is a key limitation (Hinlo et al. 2017). Future studies may look to test for pre‐filtration (e.g., Turner et al. 2014), pooling of extractions from multiple smaller volume samples, or implement passive filtration methods (Stevens et al. 2024). Moreover, during the harvesting when less water is available, eDNA approaches may also create bias due to the increased inhibitors within the water samples, putatively impacting amplification and thus biodiversity recovery. Thus, additional soil and swabbing, or washing of rice plants for eDNA, could possibly maximize detection. Still, our eDNA‐based approach detected a broad range of taxonomic groups with only limited sampling effort and without requiring extensive taxonomic expertise. Thus, our method provides a fast and effective tool for the regular monitoring of native and non‐native pests in rice fields, and with developing technology, it will be easy to use for farmers. Furthermore, incorporating multiple sample types (e.g., water, soil, leaf swabs), using group‐specific primers, and increasing the number of samples could further maximize the effectiveness of this monitoring approach.

## 5 Conclusions

Environmental DNA metabarcoding is an effective tool in revealing the diversity and composition of communities in rice fields across Taiwan. Through this study, a wide range of taxa were detected in a limited number of sampling events, providing valuable insights into the pest community. Three detected pests (*Chilo suppressalis*, *Coptotermes formosanus* , and *Pomacea canaliculata* ) are highly concerning. Notably, the presence of non‐native *Pomacea canaliculata* dominates over native snails, raising concerns about the impact on native ecosystems and agricultural productivity. Furthermore, the detection of disease vectors like the Japanese Encephalitis virus from the stagnant rice field water is a rising concern for human health. These findings are particularly important for sensitive agricultural systems where ecological and biological processes are becoming increasingly recognized as essential for maintaining their ecological services. Overall, this study highlights the transformative potential of eDNA metabarcoding for rapidly evaluating dynamic biological and environmental systems.

## Author Contributions

**Pritam Banerjee:** conceptualization (equal), data curation (equal), formal analysis (equal), funding acquisition (equal), investigation (equal), methodology (equal), software (equal), validation (equal), visualization (equal), writing – original draft (equal), writing – review and editing (equal). **Gobinda Dey:** conceptualization (equal), investigation (equal), methodology (equal), validation (equal), writing – review and editing (equal). **Kathryn A. Stewart:** data curation (equal), methodology (equal), validation (equal), writing – review and editing (equal). **Matthew A. Barnes:** data curation (equal), validation (equal), writing – review and editing (equal). **Md. Taharia:** data curation (equal), formal analysis (equal), methodology (equal), writing – review and editing (equal). **Mathew Seymour:** data curation (equal), methodology (equal), validation (equal), writing – review and editing (equal). **Chin‐Wen Wang:** data curation (equal), investigation (equal), methodology (equal), writing – review and editing (equal). **Raju Kumar Sharma:** data curation (equal), investigation (equal), validation (equal), writing – review and editing (equal). **Jyoti Prakash Maity:** data curation (equal), investigation (equal), supervision (equal), writing – review and editing (equal). **Chien‐Yen Chen:** conceptualization (equal), funding acquisition (equal), project administration (equal), resources (equal), supervision (equal), validation (equal), writing – review and editing (equal).

## Funding

This work was supported by Chiayi Christian Hospital (CYCH‐CCU‐2023‐01), and National Science and Technology Council (NSTC: 112‐2116‐M‐194‐010).

## Conflicts of Interest

The authors declare no conflicts of interest.

## Data Availability

Data sharing supporting the study is openly available in Dryad (https://doi.org/10.5061/dryad.mw6m9069f).
